# The association between organophosphate pesticide residue exposure and non-alcoholic fatty liver disease: A systematic review

**DOI:** 10.1016/j.toxrep.2026.102216

**Published:** 2026-02-03

**Authors:** Bahare Mohamadi, Mohammadreza Tolou Ostadan, Samira Shokri, Alireza Bakhtiyari, Rokhsana Rasooli, Parisa Sadighara, Saeed Aghebat-Bekheir

**Affiliations:** a Student's Scientific Research Center, Division of Food Safety and Hygiene, Department of Environmental Health Engineering, School of Public Health, Tehran University of Medical Sciences, Tehran, Iran; b Department of Pediatric Surgery, Bahrami Hospital, Tehran University of Medical Sciences, Tehran, Iran; c Nutritional Health Research Center, Lorestan University of Medical Sciences, Khorramabad, Iran; d Department of Pharmacology, Faculty of Veterinary Medicine, University of Tehran, Tehran, Iran; e Department of Environmental Health Engineering, Division of Food Safety and Hygiene, School of Public Health, Tehran University of Medical Sciences, Tehran, Iran; f Department of Toxicology & Pharmacology, School of Pharmacy, Tehran University of Medical Sciences, Tehran, Iran

**Keywords:** Fatty liver, Nutrition, Pesticides, Poisoning, Organophosphate

## Abstract

Non-alcoholic fatty liver disease (NAFLD) is a chronic disease that has had a significant prevalence in recent decades. Various factors contribute to the disease, with diet being one of the most important. In recent decades, several studies have reported a correlation between agricultural pesticide residues and NAFLD. This systematic review aimed to discuss scientific findings and analyze evidence of the association between organophosphate pesticide residues and NAFLD. To achieve this, relevant keywords were identified, and a search protocol was established in databases over the past decade to facilitate article retrieval. Finally, a total of 314 articles were identified through the search, of which 21 met the inclusion criteria and were selected for this review. This review identified a diverse range of OPs and their metabolites concerning NAFLD. Glyphosate and its formulations (such as Roundup) were the most studied OPs. The key OPE metabolites most frequently studied were BDCIPP, BCIPHIPP, DPHP, and BCEP. In addition, pesticides such as triphenyl phosphate (TPHP), trichlorofon, tris(1-chloro-2-propyl) phosphate (TCPP), tris(2-chloroethyl) phosphate (TCEP), and tri-ortho-cresyl phosphate (TOCP) were also investigated. The primary method for assessing OPs exposure involved measuring urinary metabolites. We discuss the evidence for the correlation between exposure to OPs and NAFLD, as well as the factors that influence it.

## Highlights

• There is a link between exposure to OPs and NAFLD.
• Demographic characteristics influence the severity of liver complications caused by OPs.
• The reference dose of OPs leading to NAFLD remains a knowledge gap.
• Diet can be a strategy to reduce NAFLD caused by OPs.
• Identifying the metabolic pathways of OPs is essential for defining prevention strategies.

## Graphical Abstract

**Figure:**
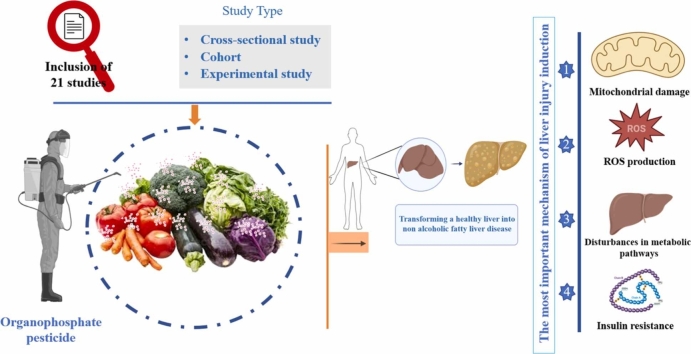

## 1 Introduction

Non-alcoholic fatty liver disease (NAFLD) is a group of metabolic liver diseases that lead to the accumulation of lipid in the liver [1]. Damage such as cell death, inflammation, and fibrosis, which can lead to liver cirrhosis and liver cancer, is a serious consequence of NAFLD [2]. Recently, the term metabolic dysfunction-associated fatty liver disease (MAFLD) was coined, referring to the significance of the metabolic dysregulation in the etiology of fatty liver disease. In recent decades, due to various factors such as lifestyle that has led to an increase in obesity, NAFLD has increased significantly, with its prevalence reported to be up to 25 % among adults [3]. Therefore, NAFLD is one of the most common liver-related diseases in the world [4]. In addition, NAFLD can provide the basis for the development of diseases of other organs and disorders, including cardiovascular diseases [5]. This disease can also be introduced as a silent disease because people with NAFLD do not have specific symptoms and only become aware of the disease when it progresses and severe liver damage occurs [3]. There is no approved drug for NAFLD [6] and that current treatments rely on dietary modification and lifestyle changes [7], understanding the factors that cause this disease and strategies for its prevention are the useful available options [3].

Previous studies have reported a relationship between diet and NAFLD, and dietary changes have been introduced as a method for controlling this disease [8]. Therefore, diet is one of the most critical factors related to NAFLD that should be taken into consideration. However, the fundamental question is which food characteristics could be important in the occurrence or control of this disease. Romero-Gómez et al. identified the Mediterranean diet, characterized by reduced carbohydrate intake and increased consumption of monounsaturated fatty acids and omega-3, as the best nutritional pattern for controlling NAFLD, which can prevent the disease even without exercise [9]. In addition to the Mediterranean diet, Moor et al. reported a vegetarian, high-protein diet and intermittent fasting as available options for the prevention and treatment of NAFLD due to their metabolic effects [6]. Lujan et al. reported that a diet rich in saturated fats, sugars, and micronutrients plays a crucial role in the development and progression of NAFLD [7]. Therefore, most previous studies include evidence of the metabolic impact of nutrition on NAFLD.

Population growth and urbanization in recent decades have led to an increase in food demand, a significant factor in agricultural development and the use of fertilizers and pesticides to maintain farm product quantities [10]. The increase in the use of pesticides in recent decades to protect agricultural products against insects and fungi has been evident, with approximately two million tons of these chemicals used in global agriculture annually [11], [12]. Organophosphates are the most widely used type of pesticide. Especially after the ban on organochlorine pesticides (OCs) in most parts of the world, the use of organophosphate pesticides (OPs) has increased dramatically, and today they account for approximately 40 % of the pesticide market [13], [14]. The use of organophosphates as pesticides is steadily growing due to several factors, including their rapid and potent effectiveness, broad spectrum of activity, relative environmental degradability compared to organochlorines (OCs), low cost, and wide availability [15], [16]. It has been proven that acute OPs poisoning in humans occurs due to the compound's ability to inhibit acetylcholinesterase activity. Organophosphates are particularly suspected of affecting NAFLD due to their lipophilic nature, their known effect on oxidative stress, or acetylcholinesterase, which can have metabolic effects. Previous studies have evaluated the relationship between OPs and NAFLD, and analyzing their findings can provide a clear perspective on this issue. This study aimed to comprehensively review scientific findings about the impact of OPs, as one of the most widely used pesticides, on the incidence and progression of NAFLD. Also, the effect of different concentrations of OPs residue in agricultural products on the progression of NAFLD was another question of this review.

## 2 Method

### 2.1 Search of the literature

**Fig. 1:**
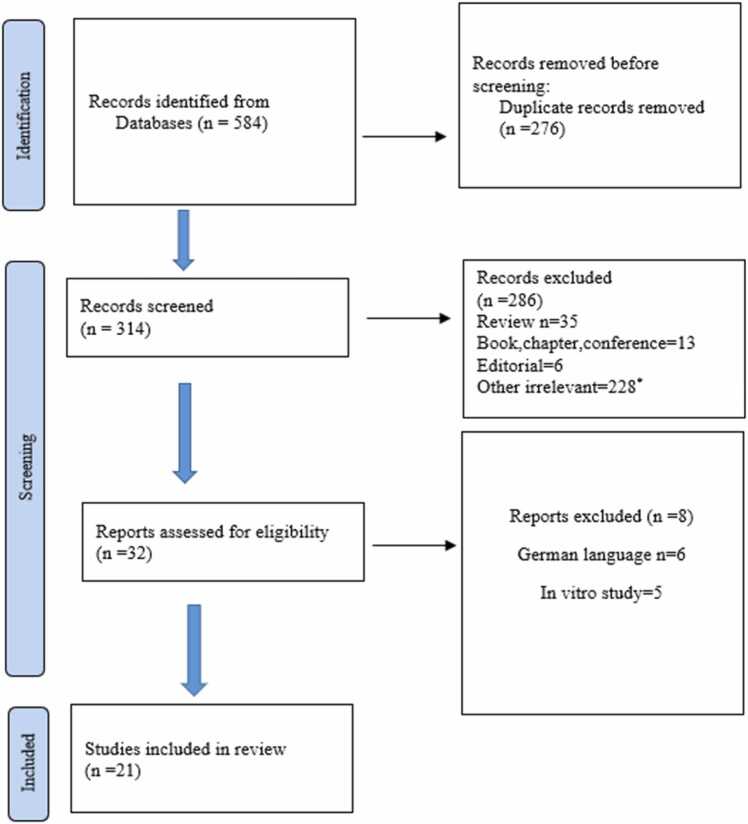
Study search process. Flow diagram showing the systematic search and selection process. It illustrates the number of records identified through database searching and other sources, duplicates removed, records screened, full-text articles assessed for eligibility, articles excluded with reasons, and studies included in the final review [17].

This study was conducted based on the 27-part Prisma checklist. This checklist ensures that we do not miss any cases. To prevent bias, each step was performed by at least two authors. First, keywords were selected in consultation with team members. Two databases, Scopus and PubMed, were selected. The search strings were used as follows: (“Non-alcoholic fatty liver disease” OR “Non-alcoholic fatty liver” OR “Nonalcoholic fatty liver” OR “Nonalcoholic fatty liver” OR “Fatty liver” OR (NAFL) OR (NAFLDs) OR (NAFLD) OR (FLD) OR Steatohepatitis OR (NASH) OR “Nonalcoholic steatohepatitis” OR “Non-alcoholic steatohepatitis” AND Organophosphates OR Organophosphat OR)OP( OR (OPs) OR Organophosphorus OR “Organophosphorus compounds” OR Glyphosate OR glyphosate OR “N-(phosphonomethyl)glycine”). (Accessed March 01, 2025). Although the mechanism differs, we include glyphosate as a structural organophosphorus compound. This strategy resulted in 314 publications, from which duplicates were removed (Fig. 1).

### 2.2 Eligibility criteria

The final papers were selected based on the eligibility criteria, specifically those studies that investigated the effect of OPs on NAFLD. Hence, all studies that (1) only mentioned NAFLD in a specific community or (2) investigated other factors affecting this disease (like lifestyle or diet) were excluded. Furthermore, articles concerning agriculture, environmental, hydrology, analytical method like detection of pesticides, diagnosis/prevalence/treatment for NAFLD, diagnosis/treatment of liver disease, nutrition studies and nutrition effect on liver metabolism, obesity, organophosphates and other disease, other pesticides or chemical contaminants were excluded.

### 2.3 Study selection

The literature was independently screened by all authors based on the criteria mentioned above. After the initial screening of the titles, 32 studies were selected (Fig. 1). Finally, 21 articles were selected for this review based on PRISMA guidelines.

### 2.4 Extraction of data

Data from totally eligible articles were extracted into a pre-defined data extraction file. The evidence of correlation between OPs and NAFLD was extracted based on the content of the eligible papers.

## 3 Results

**Fig. 2:**
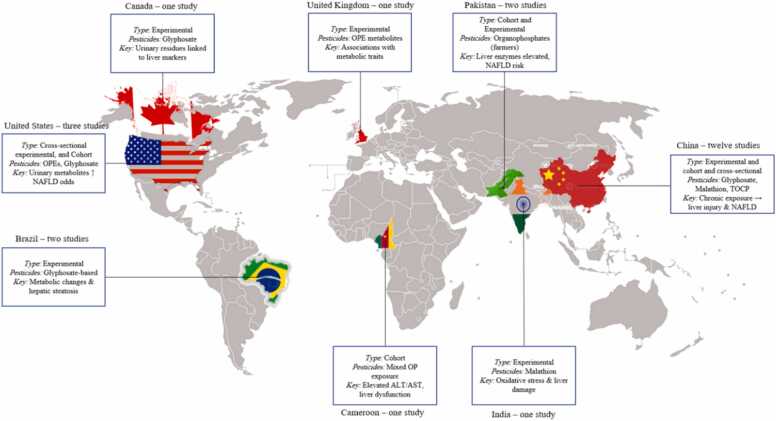
The geographical distribution of studies (Two studies were conducted jointly by Pakistan and China/and another one involved collaboration between Pakistan and Cameroon.).

The geographical distribution of included studies revealed a significant concentration of research in Asia, with a notable origin from China (12 studies), followed by Pakistan (2 studies) and India (1 study). Studies from North America were primarily from the United States (3 studies) and Canada with one study. A limited number of studies originated from Europe (the United Kingdom, with one study), South America (Brazil, with two studies), and Africa (Cameroon), also with one study (Fig. 2).

This systematic review identified a diverse range of organophosphate pesticides and their metabolites that have been studied in relation to NAFLD. Glyphosate and its formulations (such as Roundup) have been the most studied, particularly in experimental studies. Several studies, especially cross-sectional ones, have focused on a mixture of organophosphate esters (OPEs) and their metabolites. The key OPE metabolites most frequently studied were BDCIPP, BCIPHIPP, DPHP, and BCEP. In addition, pesticides such as triphenyl phosphate (TPHP), trichlorofon, tris(1-chloro-2-propyl) phosphate (TCPP), tris(2-chloroethyl) phosphate (TCEP), and tri-ortho-cresyl phosphate (TOCP) were also investigated. Some pesticides, like malathion and chlorpyrifos, have been reported in both cohort and experimental studies.

The route of human exposure was mostly considered environmental exposure. In cross-sectional and cohort studies, the primary method for assessing OP exposure involved measuring urinary metabolites. Methods such as LC-MS/MS, UPLC–MS/MS, and IC-MS/MS were commonly used to quantify these metabolites from spot urine samples, in conjunction with spectrometry. HPLC-MS, GC-MS, and ELISA, as well as NHANES data, were also utilized in fewer studies. For experimental animal studies, OPs exposure was controlled through various routes, particularly oral gavage and drinking water. Doses were specified, and their durations varied from acute to chronic. Additionally, waterborne exposure was used for aquatic animal models such as crucian carp and zebrafish. In experimental studies, a wide range of methods were employed to assess organophosphates levels or their impact. To quantify organophosphates or their metabolites, studies utilized sophisticated mass spectrometry-based techniques such as Tandem Mass Tag-LC-MS/MS, UPLC-MS/MS, and GC-MS/MS to measure their levels in serum, liver, and other tissues. LC-MS/MS and GC-MS methods were also employed in these studies. Beyond direct measurement of the compounds, a wide array of methods focused on the biological and physiological responses to exposure. This included histopathology (using stains such as H&E and Oil Red O), biochemical assays, and molecular techniques (including qPCR, RNA-Seq [transcriptome sequencing], Western blot, and ELISA), which were reported in the included studies. Other methods included immunohistochemistry, immunofluorescence, 16S rRNA, and 1 H NMR for metabolomics. Moreover, physiological and metabolic assessments, as well as body weight, are also evaluated.

The diagnosis of NAFLD or metabolic dysfunction-associated fatty liver disease (MAFLD) in the included studies relied on non-invasive scoring systems and imaging techniques. For human studies, USFLI, FLI, and HIS are often combined with metabolic criteria. A comprehensive panel of liver-related biomarkers was frequently assessed from blood samples, including liver function enzymes (ALT, AST, ALP, GGT), as well as advanced imaging methods, such as ultrasound and MRI. Glycemic markers such as FPG, insulin, cardiometabolic traits, and TC, TG, LDL-C, HDL-C, SBP, DBP, and weight were assessed in some reports.

In experimental animal studies, the focus was on histopathological changes in the liver, including hepatic steatosis, NAS, liver index, body weight, liver weight, food/water intake, glucose tolerance, and insulin sensitivity. Key biochemical and molecular outcomes, mostly measured, included ALT and AST, lipid accumulation, liver TG, TC, FFA, along with associated gene expression, protein content, oxidative stress markers such as MDA, ROS, and the activity of antioxidant enzymes (SOD, CAT, GST, GPx).

Overall, cross-sectional, cohort, and experimental studies indicate a significant correlation between organophosphate exposure and the development or progression of NAFLD/MAFLD.

## 4 Discussion

### 4.1 The importance of NAFLD and the role of organophosphates

Considering various causative factors, NAFLD is known as a complex disease, but some underlying diseases such as obesity, type 2 diabetes, and insulin resistance can lead to NAFLD [38], [39]. In addition, the lack of early symptoms caused by NAFLD and its association with other causes of mortality, such as cardiovascular diseases [40], leads to the underreporting of many cases of this disease, especially in poor and developing countries. In this situation, global data indicate a significant prevalence of 38 % and an annual incidence of 4613 cases per 100,000 people [40]. Data show that more than 1 billion of the world's population is affected by NAFLD, and the proportion of affected individuals has increased in recent decades, although the geographical distribution of the disease is not the same in different countries [41].

While NAFLD is a silent disease, lifestyle factors, including physical activity and nutrition have significantly correlation with its incident or control [42]. Several studies have reported the positive effects of weight loss on reversing steatosis and fibrosis [43], [44], and several studies have suggested certain dietary patterns, such as the Mediterranean diet and high-protein diets, for the prevention and control of NAFLD [45], [46]. However, the biochemical nature and potential contaminants in various foods have received less attention. Considering that in a category, NAFLD can be divided into two types including NAFLD with healthy metabolism and NAFLD with metabolic dysfunction [47], studies related to diet and NAFLD could be an answer to the concerns related to NAFLD with healthy metabolism. In particular, metabolically healthy obesity, which is a common term for obese individuals without metabolic abnormalities [48], is known as a risk factor for incident NAFLD because in various studies, the incidence of NAFLD in metabolically healthy obesity individuals has been reported to be significantly higher [49]. Therefore, diets recommended for reducing NAFLD that aim to reduce obesity can be effective in controlling metabolically healthy NAFLD. However, various factors, including agricultural toxins (pesticides) or food spoilage contamination that can lead to metabolic dysfunction, can be effective in increasing the incidence of NAFLD with metabolic dysfunction.

Pesticides are a primary factor in agricultural production and have been used increasingly in recent decades, particularly in developing countries [50], [51]. According to the results of numerous toxicological studies, pesticides are among the primary concerns related to metabolic disorders [52]. The importance of exposure to OPs in the incidence of NAFLD becomes clear by considering the function of this group of pesticides as acetylcholinesterase (AChE) inhibitors and the disruption of the function of peripheral and central nervous systems, muscles, liver, pancreas, and brain [53] of the target pest. In addition, the importance of lipid and carbohydrate metabolism in incidence of NAFLD [54] makes factors that disrupt these metabolisms potentially associated with NAFLD with metabolic dysfunction. Therefore, OPs can be considered as one of the potential factors for the incidence and progression of NAFLD due to the disruption of enzymatic pathways involved in the metabolism of carbohydrates, fats, and proteins in the cytoplasm, mitochondria, and peroxisomes [55]. This hypothesis can be examined by evaluating the results of previous studies on the incidence or progression of NAFLD in the exposure to OPs, which is discussed below.

### 4.2 Reported evidence from animal studies

Numerous evidences of lipid accumulation in the liver of studied samples such as fish confirm the correlation between exposure to OPs and the occurrence of liver disorders including NAFLD [35], [36], [37]. The concentration of the pollutant is an essential factor in the severity of NAFLD in chronic exposure to OPs. It is naturally expected that exposure to higher concentrations of OPs will lead to more severe liver complications. Although evidence from patient studies on this aspect of the issue is not available, Pandey et al. (2019) showed that mice receiving higher concentrations of the pollutant exhibited more severe multiorgan inflammation, scarring, and liver dysfunction [27]. In addition, considering liver diseases, including NAFLD, is essential in defining exposure standards or limits because there is evidence that low concentrations, even within toxicological limits, can contribute to the occurrence of NAFLD. Romualdo et al., 2023, by studying male mice in a controlled laboratory environment, showed that exposure to one of the types of OPs at a dose within the toxicological range was able to cause disorders mainly in the liver and intestinal axes involved in the development of NAFLD [28]. However, the exposure time can also affect the severity of the effects, such that the occurrence of NAFLD in studied mice during six months of exposure to glyphosate was reported only at concentrations higher than 50 mg/kg [29]. In addition, acute exposure to high concentrations of OPs can also be considered as a factor affecting the occurrence of NAFLD. Li and Wu. In 2024, a study described the industrial applications of OPs and investigated the exposure of mice to Tri-ortho-cresyl phosphate (TOCP) at a concentration of 800 mg/kg, reporting evidence of NAFLD and hepatic steatosis in the studied samples [30].

Results of a study on mice showed that exposure to OPs could reduce elevated blood glucose levels without altering basal glucose levels [56]. Additionally, exposure to high levels of organophosphate flame retardants can disrupt the tricarboxylic acid cycle in men, which can result in NAFLD [57]. Tris(2-chloroethyl) phosphate (TCEP) has been observed to increase malondialdehyde levels in mice, thereby inducing oxidative stress and endocrine disruption [58]. It has been demonstrated that malathion elevates insulin resistance, inflammation, and steatosis in rats through mechanisms involving oxidative stress [59]. Reports have shown that exposure to tri-n-butyl phosphate (TNBP) and tris(2-butoxyethyl) phosphate (TBOEP)is associated with oxidative stress and pathological damage in mice [60]. This risk can include carcinogenic, mutagenic, and chronic disease risks. Also, more evidence is needed in specific cases, such as pregnancy and the effect of pollutant exposure on fetal health. For example, Novbatova et al. reported changes in liver protein in pregnant mice exposed to glyphosate before conception, which were identified as being associated with liver diseases, including NAFLD [26].

### 4.3 Reported evidence from human case studies

Several studies have suggested a possible link between organophosphate stress (OPEs) with MAFLD and NAFLD in humans, but this has not yet been definitively confirmed. However, the evidence and findings of previous studies can be discussed. A study of 2618 cases from 2011 to 2018 in China showed a positive and significant correlation between OPEs metabolites and NAFLD and MAFLD, indicating a higher probability of developing MAFLD and NAFLD in individuals with elevated concentrations of OPEs metabolites [18]. But this study attributes the concentration of OPEs metabolites to diet and concludes that a healthier diet may lead to lower levels of certain OPEs metabolites, which reduces the risk of developing NAFLD and MAFLD [18]. This important finding can be considered as evidence of the correlation between OPs concentrations in agricultural products that consist diet with the possibility of developing NAFLD and MAFLD.

Although evidence is available on the association between exposure to OPs and incident NAFLD, the influence of general factors such as demographic characteristics on the incidence of this disease should also be considered [61]. For example, a study of NAFLD cases over a 17-year period to 2016 in the United States showed that characteristics such as obesity, age, sex, and race were effective in the incidence of NAFLD, such that cases were more common in older age, men, obese individuals, and people of Oriental and Caucasian races [62]. Therefore, exposure to certain concentrations of OPs could cause a higher risk of incident and progression of NAFLD in susceptible groups. For example, a study of 1102 cases in the United States from 2011 to 2014 showed that environmental OPE exposure was associated with an increased risk of NAFLD in men, especially those over 60 years of age or with low testosterone levels, but this effect was not observed in other women studied except in postmenopausal women [3]. Although higher probability of developing NAFLD in men due to exposure to OPs has been reported in other studies [21], However, there are also reports of a more pronounced correlation between OPs and NAFLD in women than in men, indicating the effect of race on OPs-related NAFLD [19]. Therefore, although a positive correlation between OPs exposure and the occurrence of NAFLD has been reported in various studies (Table 1), several secondary factors can be considered in this issue.

**Table 1:** The extracted data according to the protocol.

**Study Type / Route/Duration /dose of exposure**	**Type of organophosphate and its metabolites**	**Measurement Method/ Biomarkers/Outcome Measured**	**Main Findings**	**Reference/country**
cross-sectional observational study/ Environmental exposure, urinary metabolites	OPEs metabolites mixture and three individual metabolites [i.e., bis(1,3-dichloro-2-propyl) phosphate (BDCIPP), bis(2-chloroethyl) phosphate, and diphenyl phosphate] were significantly and positively associated with NAFLD and MAFLD (P-trend<0.001),	Urinary OPEs metabolites measurement using spot urine samples from participants of the NHANES 2011–2018 survey cycles/ liver-related biomarkers from blood, ultrasound elastography (Controlled Attenuation Parameter, liver stiffness measurement)/ Primary outcomes: Hepatic steatosis diagnosed using USFLI ≥ 30 NAFLD defined as USFLI ≥ 30 in absence of viral hepatitis and excessive alcohol intake MAFLD defined as USFLI ≥ 30 + (BMI ≥ 25 kg/m² or diabetes or metabolic dysregulation) Secondary outcomes (biomarkers): Glycemic markers: FPG, insulin, 2h-OGTT, HbA1c, HOMA-IR Liver function enzymes: ALT, AST, ALP, GGT Liver ultrasound markers: CAP (steatosis), LSM (fibrosis) Cardiometabolic traits: TC, TG, LDL-C, HDL-C, SBP, DBP, waist circumference, weight	Positive associations between several urinary OPEs (especially BDCIPP) and NAFLD / MAFLD Diet quality scores negatively associated with NAFLD / MAFLD and with urinary OPEs no interaction was observed between diet quality and OPEs, participants with both low BDCIPP levels and high diet quality had the lowest odds of MAFLD and NAFLD.	China [18]
cross-sectional/ Single spot urine sample (0.4 mL); environmental subsample of NHANES participants aged ≥ 20; two 2-year cycles	5 OPE metabolites: DPHP, BDCPP, BCPP, BCEP, DBUP	LC-MS/MS for quantification of urinary OPE metabolites/US FLI, which has been shown to be more accurate than the Fatty Liver Index in the US population. FLI < 30 excludes fatty liver, the specificity is 52 % (sensitivity 83 %). FLI ≥ 60 determines fatty liver with a sensitivity of 69 %, (specificity 77 %) Race/ethnicity, age, waist circumference, GGT, rapid insulin, and glucose are all used to generate the US FLI	BCEP and BDCPP positively associated with NAFLD in men; strongest in men < 60 or with low TT; OPE index also associated with NAFLD in postmenopausal women these correlations were weaker in women.	China [3]
Cross-sectional study (NHANES 2013–2016)/ Environmental exposure; measured by spot urine	Glyphosate (GLY)	Urinary glyphosate (uGLY), analyzed by IC-MS/MS with isotope dilution quantification/ Fatty Liver Index (FLI), calculated from TG, BMI, GGT, and WC; subgroup analyses for sex, age, comorbidities	positive linear association between increased level of GLY in urine and the FLI (β=2.16, 95 % CI: 0.71–3.61); stronger in females, 40–60 y/o, other ethnicities, borderline diabetes, and without hypertension	US [19]
cross-sectional series/ Environmental exposure by general population; chronic, based on NHANES data (multiple cycles)	Organophosphate metabolites (in mixture with pyrethroids and herbicides)	Urinary OP metabolites, Weighted Quantile Sum (WQS) regression using urinary biomarkers from NHANES data analysis; data from NHANES/ Diagnosis of NAFLD using US Fatty Liver Index (USFLI) and Hepatic Steatosis Index (HSI)	Significant positive association between urinary OP metabolites and NAFLD (OR: 1.32, 95 % CI: 1.15–1.51); stronger in individuals with healthy lifestyle (HLS 3–4)	China )data availability of NHANES( [20]
cross-sectional study/ Environmental exposure; measured via urine; duration not specified	organophosphate esters(fifteen urinary OPE metabolites, including bis(1-chloro-2-propyl) 1-hydroxy2-propyl phosphate (BCIPHIPP), 4- bis(1,3-dichloro-2-propyl) phosphate (BDCIPP), etc.	UPLC–MS/MS (ultra-performance liquid chromatography–tandem mass spectrometry)/ Ultrasound for liver steatosis + metabolic criteria (BMI, T2DM, dysregulation)/ Urinary OPE metabolites; Diagnosis of MAFLD (by ultrasound and metabolic criteria)	Higher urinary levels of BCIPHIPP and BDCIPP associated with increased odds of MAFLD, especially in men; significant gender differences observed in associations.	China [21]
cross-sectional study/ Urinary levels;No specific dose or duration mentioned	chlorpyrifos	fat fraction (FF) values by MRI/ LC-MS/ Urinary levels of Chlorpyrifos/Fat Fraction (FF) by MRI for fatty liver diagnosis	Chlorpyrifos levels are positively correlated with the severity of fatty liver disease (FLD). Chlorpyrifos may serve as predictor for FLD.	China [22]
Cohort/ inhabitants of the pesticide-sprayed areas for at least six months and were exposed to OPPs directly (farmers and sprayers) or indirectly (people living in the spraying areas) during the period.	OPPs (malathion, parathion, chlorpyrifos,	Blood samples/ GC-MS for OPP residues; ELISA and spectro- photometry for bioch- emical para meters/ AST, ALT, LDH, γGT, ALP, bilirubin	A significant elevation in liver enzymes (ALT, AST, LDH, ALP, bilirubin) was observed in OPPs-exposed individuals, suggesting liver dysfunction potentially linked to NAFLD. The findings indicate that chronic low-dose OPPs exposure may contribute to the development of NAFLD either directly or through OPP-induced type 2 diabetes and metabolic syndrome. e.) the direct consequences of T2D may lead to the progressive onset of MetS, nonalcoholic fatty liver disease and renal failure (	Pakistan and Cameroon [23]
Cohort/ Ingestion of glyphosate by diet (non-agricultural exposure)/ Urinary excretion, measured over a period of time from 2012 to 2018, at a dose of 0.1 ppb (50 ng/L glyphosate equivalent daily intake)	Glyphosate	HPLC coupled with Mass Spectrometry / biopsy/ Glyphosate, AMPA, glyphosate residue in urine/ AST, ALT, Triglycerides, Cholesterol Levels	Elevated glyphosate excretion was associated with NASH and advanced liver fibrosis. Women had higher glyphosate excretion than men. Glyphosate exposure increased with liver fibrosis stages. Glyphosate residue was significantly higher in NASH and advanced fibrosis compared to those without NASH and lower fibrosis stages.	USA
Experimental (rat)/ Oral (drinking water) for 2 years with a dose of 50 ng/L glyphosate equivalent (∼4 ng/kg body weight/day)	Roundup herbicide (Glyphosate-based)	Tandem Mass Tag-LC-MS/MS, UPLC-MS/MS, GC-MS /Anatomorphological changes, Blood/Urine biochemical analysis, Transcriptome profiling, Multiomic analysis/ Liver Proteome and Metabolome (anatomorphological changes), Histopathological Changes, biochemical changes (blood/urine), changes in gene expression (transcriptome)	metabolite alterations associated with hallmarks of hepatotoxicity such as γ-glutamyl dipeptides, acylcarnitines, and proline derivatives, Significant alterations in liver proteome and metabolome, overlap with biomarkers of NAFLD and NASH progression correlates with hepatotoxicity and biochemical changes in liver resulting from chronic ultra-low dose GBH exposure	United Kingdom [25]
Experimental study (mice)/ oral (gavage), daily for 10 weeks before conception; dose: 2 mg/kg	Glyphosate	LC-MS/MS for liver and ovarian proteomics; Histology; Morphometrics (AGD, body weight, puberty onset, follicle counts); PCR for sex determination/ Altered liver proteome: pathways including glutathione metabolism, oxidative phosphorylation, thermogenesis, and NAFLD; ovarian protein changes; body weight	Maternal pre-conceptional exposure to glyphosate altered the hepatic proteome of female offspring, including pathways related to metabolic function and NAFLD. No gross phenotypic changes in liver weight, puberty timing, AGD, or sex ratio. Increase in male body weight and minor reduction in testes/body weight ratio.	USA [26]
Experimental study (mice)/ Oral, daily for 14 days; doses: 0, 5, 10, 25, 50, 100, 250 mg/kg bw/d	Roundup )Glyphosate(	qPCR (gene expression), histopathology, liver index, CRP assay/ Liver index, body weight, liver weight, CRP (liver), IL-1β, TNF-α, IL-6 (liver & adipose), PTGS (COX-2) expression, histological changes (vacuoles, fibrosis, glycogen depletion)	Dose-dependent increase in IL-1β, TNF-α, IL-6 in liver; elevation at 100 and 250 mg/kg bw. PTGS increased from 50 mg/kg. Liver CRP elevated. Histology showed vacuoles, fibrotic tissue, and glycogen loss, indicating inflammation and early-stage NAFLD. Body weight reduction observed at higher doses. Adipose tissue showed IL-6 increase and altered cytokine response.	India [27]
Experimental study (mice)/ Oral gavage, 5 × /week for 6 months alongside western diet (high fat/sugar)	Glyphosate (0.05 or 5 mg/kg/day), alone or in combination with 2,4-D	Histology (H&E, Sirius Red), gene expression (mRNA-seq), ELISA, MDA, antioxidant enzymes, GTT, cholesterol, ALT, NAS score, collagen morphometry, fibrosis grade, mast cell counting/ Hepatic steatosis, fibrosis, inflammation (NAS score, lobular inflammation, CD68 + macrophages), oxidative stress (MDA, antioxidant enzymes), glucose metabolism (GTT), serum cholesterol and ALT	High-dose 2,4-D (2 mg/kg) worsened liver inflammation, increased CD68 + cells, fibrosis, and markers of oxidative stress. Glyphosate alone or in mixture did not show same effects. Herbicide mixture did not exacerbate NAFLD as 2,4-D alone. NAFLD was mainly driven by 2,4-D exposure.	Brazil [28]
Experimental (mice)/ Oral gavage; 6 months; 0.05, 5, or 50 mg/kg/day	glyphosate	Histology (H&E, Sirius red), Immunohistochemistry (CD68, α-SMA, Ki67), Western blot (NF-κB p65, Nrf2), ELISA (TNF-α, IL-6), GTT, serum biochemistry (ALT, cholesterol), RNA-seq (DEGs)/ NAS (steatosis, inflammation, hypertrophy), collagen content, CD68 + macrophages, TNF-α, IL-6, Nrf2, MDA, SOD, catalase, gene expression (oxidative stress, inflammation, cell cycle)	Glyphosate at 50 mg/kg increased liver inflammation markers (CD68 +, TNF-α, IL-6), reduced antioxidant Nrf2, increased lipid peroxidation (MDA), altered gene expression; but did not aggravate obesity, steatosis, or fibrosis.may predispose to NAFLD-to NASH progression upon long-term exposure	Brazil [29]
Experimental (mice)/ Oral, single dose of TOCP (800 mg/kg, p.o.) measurements at 6 h, 1 day, and 7 days	Tri-ortho-cresyl phosphate	Histopathology (HE and Oil Red O staining), serum biochemistry (AST, ALP, TP), Western blot/ Hepatic steatosis, lipid droplets (LDs), TG in serum/liver, GRP78, CHOP, XBP1s, p-mTOR, SREBP1c	TOCP induced acute hepatic steatosis in mice by increasing LDs and triglycerides in liver and serum. It activated ER stress (GRP78, CHOP, XBP1s) and mTOR signaling (p-mTOR), promoting de novo lipogenesis. These effects were significantly inhibited by ER stress inhibitor (4-PBA) and mTOR inhibitor (rapamycin) that shows involvement of both pathways.	China [30]
Experimental (mice)/ Oral gavage (intragastric), 60 mg/kg body weight, daily for 9 weeks	Tris (2-chloroethyl) phosphate (TCEP)	Serum biochemistry, liver histopathology, 16S rRNA sequencing, Western blot/ Body weight, food/water intake, WAT/BAT index, plasma and liver TG, NEFA, LDLC, HDLC, ALT, AST, total bile acids, liver histology, gut microbiota, expression of FXR, SHP, PPARα/γ, SREBP1c, FASN	Chronic oral exposure to TCEP induced hepatic steatosis characteristic of NAFLD, including increased liver weight, elevated hepatic triglycerides and total bile acids, and histological evidence of fat accumulation in the liver. It also led to elevated plasma ALT and AST levels, indicating liver injury. Co-administration of complex probiotics significantly ameliorated these liver-related changes by restoring lipid metabolism, reducing liver fat accumulation, improving liver enzyme levels, and modulating FXR signaling pathways involved in hepatic lipid regulation.	China [31]
Experimental (mice)/ Oral, dietary exposure; mice (0.5 or 2 mg/kg body weight); exposure duration: up to 9 weeks	chlorpyrifos	In vivo: physiological and biochemical assays, metabolic cages, histology, gene/protein expression, TEM (Tissue processing, histological examination, and transmission electron microscopy)/ Body weight, adiposity, glucose tolerance, insulin sensitivity, serum FFA and TG, liver TG, liver weight, ALT, AST	Chlorpyrifos at low doses, especially under thermoneutral conditions with high-fat diet, impairs thermogenesis, increases obesity, insulin resistance, and severity of NAFLD in mice. Effects are not due to cholinesterase inhibition.	Canada [32]
Experimental (mice)/ Oral (in utero and lactational); 10, 100, 1000 mg/kg BW for 10 weeks	triphenyl phosphate )TPHP(	Body weight, liver histology (H&E), GC-MS (fatty acids), qPCR (gene expression), 16S rRNA (microbiota), 1 H NMR (metabolomics), GTT, HOMA-IR/ Body weight, liver weight, hepatic TG, gene expression (Pparg, Cd36, Fasn, Acaca, etc.), glucose, insulin, HOMA-IR, bile acids, SCFAs	TPHP exposure promote the initialization and development of metabolic dysfunctions, including obesity, NAFLD and diabetes. The NAFLD mechanism might result from the impaired lipid and glucose homeostasis (Fetal TPHP exposure promoted obesity, NAFLD, insulin resistance; altered lipid metabolism gene expression; changed gut microbiome and fecal metabolome (↑ bile acids, SCFAs); effects were exacerbated by High-Fat Diet)	China [33]
Experimental rooster model / Oral gavage / 180 days / low, medium, and high doses	Glyphosate	GC-MS/MS for Gly levels in serum and liver; Histology (H&E, Oil Red O); RNA-Seq; Western blot; RT-qPCR, Biochemical assays/ Gly levels in serum/liver, AST, ALT, TG, TC, LDL-C, HDL-C, liver ATP, hydroxybutyrate, NAFLD score, LC3-II, p62, p-mTOR, PPARα, FABP1, CPT1A, CD36, autophagy markers (LC3-II, p62), p-mTOR, lipid droplet staining, PPARα, FABP1, CPT1A, CD36 expression	the roosters in high-dose Gly group experienced moderate NAFLD. Interestingly, the liver TG and TC contents in high-dose Gly group were evidently increased, suggesting that the exposure of high-dose Gly induced hepatic lipid accumulation with more TG and TC contents accumulated in liver tissues/ Gly caused dose-dependent hepatic steatosis and lipid accumulation, disrupted serum lipid profile, inhibited autophagy via mTORC1 pathway, decreased fatty acid oxidation by downregulating PPARα and its target genes; Feno (PPARα agonist) alleviated Gly-induced effects; NAFLD observed in high-dose group	China [34]
Experimental (crucian carp)/ Waterborne exposure for 30 days; doses: 0, 0.5, 1.0, 2.0, 4.0 mg/L	Trichlorfon	ELISA for hepatic samples, Biochemical assays (ELISA, RIA, enzymatic methods), TEM microscopy/ Hepatic/plasma triglyceride, insulin, VLDL, ApoB100, cAMP; histological liver changes	Trichlorfon increased hepatic lipid accumulation, plasma insulin, and altered lipid metabolism pathways; observed liver ultrastructure damage	China [35]
Experimental (larval/ adult zebrafish)/ Waterborne exposure; Acute and chronic; 5, 15, 25 mg/L; Duration: 120 hpf (larvae), 90 days (adults)	tris(1-chloro-2-propyl) phosphate	Histopathology (H&E, ORO), biochemical assays (TG, T-CHO, SOD, CAT, MDA), qRT-PCR, transcriptome sequencing, immunofluorescence/ Lipid accumulation (TG, T-CHO), gene expression (Fasn, CEBPA, pparα, cpt1α, NF-κB, IL-1β, IL-22, TNF-α), oxidative stress (SOD, CAT, GPx, GST, MDA), inflammation, NAFLD, hepatocellular damage	TCPP caused lipid metabolism disruption via upregulating adipogenesis genes and downregulating β-oxidation genes; induced oxidative stress, inflammation, liver steatosis, and signs of NAFLD and potential hepatocellular carcinoma in adult zebrafish	China [36]
Experimental (rohu fish)/ Semi-static exposure to Malathion (LC50) for 96 h	Malathion	Histopathology (liver), biochemical assays (protein contents, LPO, ROS, antioxidant enzymes), DNA damage, enzyme activities/ Protein contents, LPO, ROS, AAT, AlAT, LDH, GDH, CAT, SOD, GST, DNA damage, hepatic necrosis, fatty infiltration, hemorrhage vacuolation, glycogen vacuolation, congestion, oxidative stress Protein contents, LPO, ROS, AAT, AlAT, LDH, GDH, CAT, SOD, GST, DNA damage, hepatic necrosis, fatty infiltration, hemorrhage vacuolation, glycogen vacuolation, congestion, oxidative stress Protein contents, LPO, ROS, AAT, AlAT, LDH, GDH, CAT, SOD, GST, DNA damage, hepatic necrosis, fatty infiltration, hemorrhage vacuolation, glycogen vacuolation, congestion, oxidative stress Protein contents, LPO, ROS, AAT, AlAT, LDH, GDH, CAT, SOD, GST, DNA damage, hepatic necrosis, fatty infiltration, hemorrhage vacuolation, glycogen vacuolation, congestion, oxidative stress Protein contents, LPO, ROS, AAT, AlAT, LDH, GDH, CAT, SOD, GST, DNA damage, hepatic necrosis, fatty infiltration, hemorrhage vacuolation, glycogen vacuolation, congestion, oxidative stress Protein contents, LPO, ROS, AAT, AlAT, LDH, GDH, CAT, SOD, GST, DNA damage, hepatic necrosis, fatty infiltration, hemorrhage vacuolation, glycogen vacuolation, congestion, oxidative stress	Malathion induced oxidative stress, increased ROS, LPO, and enzyme activities (AAT, AlAT, GDH, LDH), liver damage (necrosis, fatty infiltration, hemorrhage, glycogen vacuolation), and DNA damage. Suggests that Malathion is a potent hepatotoxic pesticide in fish.	Pakistan and China [37]

In addition to OPs, exposure to other chemicals can also contribute to the occurrence of NAFLD; however, a study's results showed a more significant correlation in the case of OPs [20]. In addition, exposure to OPs can lead to other metabolic disorders and complications in different organs. For example, a study of the impact of exposure to OPs in citizens of Cameroon and Pakistan showed that chronic exposure to these pesticides increased body mass index (BMI), insulin, blood glucose, dyslipidemia, hypertension, and impaired liver and kidney function in all participants, regardless of gender and age groups [23]. Therefore, the consequences of exposure to OPs in the development of NAFLD can be assessed in the context of the adverse effects of pesticides on health. Hence, to definitively determine OPs as a cause of liver dysfunction, including NAFLD, specific markers are needed. OPs metabolites that can be detected in blood and urine can serve as an indicator in definitively determining the cause of NAFLD associated with these pollutants [63]. Considering the positive correlation between pesticide metabolites and NAFLD, the levels of pesticide metabolites in urine can be used as predictors of this disease, as reported by Ma et al. (2024), who identified urinary chlorpyrifos and paraquat as biomarkers for predicting NAFLD development [22]. Additionally, increased glyphosate excretion resulting from NAFLD (24) highlights the importance of OP metabolites as a biomarker for NAFLD.

The reported effects of OPs exposure on liver function and incidence of NAFLD can be considered as a measure of the occupational health aspects of farmers and OPs-related occupations. In recent years, the occupational cancer risk of farmers due to exposure to various pesticides has been studied [64], [65]. However, the health risks associated with exposure to multiple pesticides, including OPs, in the development of diseases such as NAFLD require further study. Mills et al. identified the consumption of contaminated food products as a route of exposure to contaminants that impair liver function [24], which can be considered in the health risk assessment of polluted food. However, identifying the reference dose of exposure to these pollutants remains a fundamental question in the context of OPs and their consequences on liver function, such as NAFLD. But reports of detectable effects of very low doses of OPs, including glyphosate-based herbicides (GBH), on liver function are available [25], which could indicate a very low reference dose for these pollutants.

Numerous animal and human studies have proven the link between gut dysbiosis and NAFLD [66], [67], [68]. In NAFLD, the liver is unable to regulate and control the gut microbiota through bile acids, resulting in a disruption of the microbiota [67]. Pesticides have the potential to alter and have adverse effects on the gut microbiota. The negative effects on the gut microbiota following NAFLD have been described for these reasons. The microbiota is also affected by the negative effects of pesticides. Of course, in order to understand the mechanisms in depth and identify the species of intestinal microbiota after exposure, 16 s RNA sequencing is required [69].

The results of the review of previous studies indicate that exposure to OPs is unavoidable. Therefore, it is essential to define effective strategies, including diet-based approaches, to mitigate the negative consequences of OPs on the liver, such as the development of NAFLD. The results of the study by Yang et al. (2022) showed that dietary probiotics protect the liver against lipid metabolic disorder induced by tris(2-chloroethyl) phosphate (TCEP) through the FXR-mediated signaling pathway in TCEP-induced metabolic disorder, ultimately leading to a decrease in systemic lipid accumulation [31]. For this purpose, it is essential to identify the paths of OPs metabolites in the development of NAFLD, some of which have been presented in previous studies. Lian et al., 2023 reported evidence of glycolysis-induced autophagy inhibition, PPARα-mediated FAO inactivation, and concomitant hepatic steatosis mediated by epigenetic reprogramming of PPARα in the studied samples [34]. In addition, considering other outcomes of OPs exposure that are more easily observed in the community can help estimate effects on the liver. Wang et al. reported adverse effects of chlorpyrifos, one of the most widely used OPs, on metabolism, leading to outcomes including NAFLD, insulin resistance, and obesity [32]. Therefore, considering the obesity epidemic in the community as one of the outcomes associated with OPs exposure can be viewed as an indication of increased NAFLD.

Glyphosate induced oxidative stress in the liver, loss of mitochondrial membrane potential, and a concomitant increase in fatty acid peroxidation [70]. Similarly, Karami-Mohajeri et al. (2011) reported that OPs disrupt enzymatic pathways related to the metabolism of carbohydrates, fats and proteins in cytoplasm, mitochondria, and peroxisomes [55]. Malathion causes oxidative stress and cell death by disrupting the mitochondrial respiratory chain, decreasing ATP, increasing ROS, and activating apoptotic pathways (such as Bax/Bcl-2, Caspase-9, and cytochrome c release), also induces elevation in lipid peroxidation index, CD3 + /CD4 + and CD3 + /CD4 + percent, and pro-inflammatory cytokines [71], [72]. Low dose of triphenyl phosphate (TPP) disrupts the proteins related to the major histocompatibility complex. However, a higher dose disrupts the synthesis of unsaturated fatty acids and steroid hormones, and exacerbates lipid accumulation [73]. Also, TPP increased expression of proteins such as fatty acid-binding protein five and other related SCPs, which activate the proliferator-activated receptor pathway and disrupt lipogenesis and cholesterol metabolism, increasing the risk of obesity and metabolic disorders, and disrupts the function of mitochondria and the endoplasmic reticulum of the liver by altering the activity of cytochrome P450 enzymes. [33], [73]. Methyl parathion and chlorpyrifos cause a reduction of PON1 mRNA and immunoreactive protein and elevated inflammatory cytokines in HepG2 cells, and consequently increase the susceptibility to inflammation and oxidative stress diseases [74].

## 5 Limitation of studies

This study faces several limitations, such as significant heterogeneity among the included studies in terms of study design, exposure doses, duration, and populations. In human research, organophosphate pesticide exposure was mainly evaluated through urinary metabolites, which indicate recent exposure rather than long-term or chronic exposure.

## 6 Conclusion

In this systematic review, the correlation between exposure to OPs and the occurrence of NAFLD, the most common liver disorder, was studied. After determining the keywords and defining the search protocol, the search was conducted in March 2025. After screening based on the inclusion criteria, 21 articles were selected for inclusion in the study. The study's results showed that there is significant evidence available on the occurrence and progression of NAFLD. Glyphosate and its formulations (such as Roundup) were the most studied OPs. The key OPE metabolites most frequently studied were BDCIPP, BCIPHIPP, DPHP, and BCEP. In addition, pesticides such as triphenyl phosphate (TPHP), trichlorofon, tris(1-chloro-2-propyl) phosphate (TCPP), tris(2-chloroethyl) phosphate (TCEP), and tri-ortho-cresyl phosphate (TOCP) were also investigated. These findings indicate that OPs pesticides may promote NAFLD through a combination of oxidative stress, liver inflammation, mitochondrial dysfunction, lipid metabolism disruption, insulin resistance, and lipid accumulation. Although the correlation between exposure to OPs and the incidence of NAFLD has been confirmed, factors such as pollutant concentration, demographic characteristics, the effect of other chemical compounds, and the reference dose for the occurrence of adverse effects remain a knowledge gap, as conflicting information can be found in previous studies. Finally, identifying the pathways leading to liver disorders resulting from exposure to OPs and finding strategies to control these complications through diet can be considered in future studies. Additionally, there is a growing need for accurate research to investigate the exact impacts of these pesticides on human health.

## CRediT authorship contribution statement

**Parisa Sadighara:** Writing – review & editing, Project administration. **Rokhsana Rasooli:** Writing – original draft. **Alireza Bakhtiyari:** Writing – original draft. **Samira Shokri:** Formal analysis, Conceptualization. **aghebat bekheir saeed:** Writing – review & editing, Writing – original draft, Validation, Supervision, Project administration. **Bahare Mohamadi:** Formal analysis, Conceptualization. **Mohammadreza Tolou Ostadan:** Formal analysis, Conceptualization.

## Informed Consent/Ethical Approval

Ethical approval was not required for this systematic review, as it was based on previously published studies.

## Funding

This study received no funding.

## Declaration of Competing Interest

The authors declare that they have no known competing financial interests or personal relationships that could have appeared to influence the work reported in this paper.

## Data Availability

The data that has been used is confidential.
